# CatReNet: interactive analysis of (auto-) catalytic reaction networks

**DOI:** 10.1093/bioinformatics/btae515

**Published:** 2024-08-20

**Authors:** Daniel H Huson, Joana C Xavier, Mike A Steel

**Affiliations:** Institute for Bioinformatics and Medical Informatics, University of Tübingen, 72076 Tübingen, Germany; Department of Chemistry, Imperial College, London SW7 2BX, United Kingdom; Biomathematics Research Centre, University of Canterbury, Christchurch 8041, New Zealand

## Abstract

**Summary:**

Catalytic reaction networks serve as fundamental models for understanding biochemical systems. CatReNet is a novel software designed to facilitate interactive analysis of such networks. It offers fast and exact algorithms for computing various types of self-sustaining autocatalytic subnetworks, including so-called CAFs (constructively autocatalytic food-generated networks), RAFs (reflexively autocatalytic food-generated networks), and pseudo-RAFs. It provides dynamic visualizations to aid exploration and understanding.

**Availability and implementation:**

This open-source Java application runs on Linux, MacOS, and Windows. It is available at https://github.com/husonlab/catrenet under a GPL3 license.

## 1 Introduction

A catalytic reaction network consists of a set of ambient food molecule types and a set of reactions that transform reactants into products, usually enabled or accelerated by catalysts. One main application is in modeling aspects of early life (Xavier *et al.* 2020), others include ecological and economic niches (Cazzolla Gatti *et al.* 2020).

Mathematically, there are several different definitions of self-sustaining “collectively autocatalytic sets” as subnetworks for which all necessary reactants and catalysts are either provided in a food set or are products of reactions in the network (Kauffman 1993). In brief, a CAF (constructively autocatalytic food generated network) is a collectively autocatalytic set that arises under the assumption that any catalytic reaction can only take place in the presence of a catalyst; more generally, an RAF (reflexively autocatalytic food-generated network) allows for reactions to initially take place at a low rate provided they are later catalyzed by a downstream product. A pseudo-RAF also allows reactions to initially take place at a low rate even if some of the reactants are not yet available, as long as they (and all necessary catalysts) become available later. Precise definitions of these concepts are provided in (Hordijk *et al.* 2015, Huson *et al.* 2024).

## 2 The software

**Figure 1.**
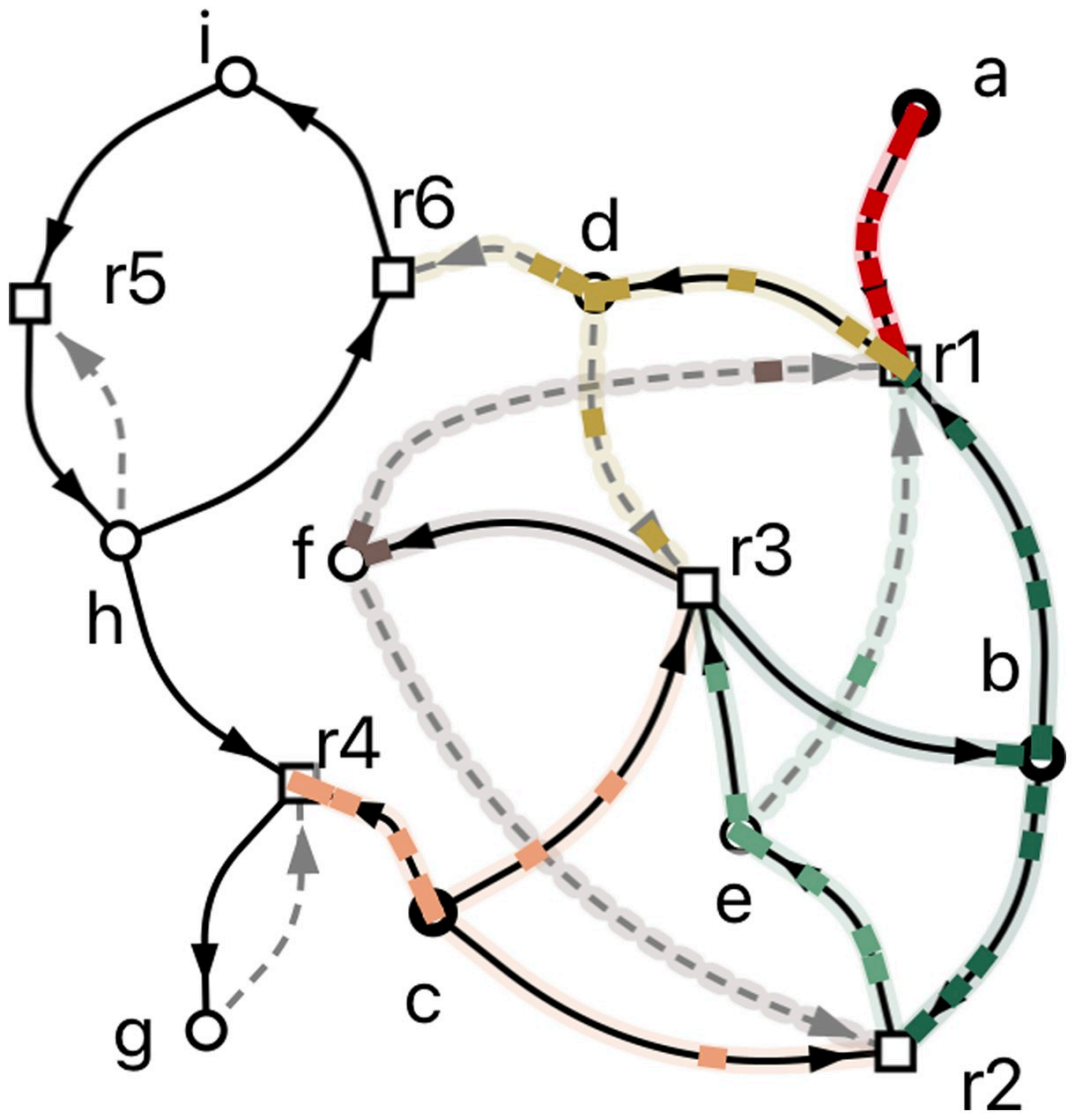
Left: Toy input with three food items a, b, and c, and six one-way reactions r1–r6. Arrows point from reactants to products and possible catalysts are listed in brackets. Right: Visualization in CatReNet, colors indicating animated maxRAF.

CatReNet is the first interactive software for computing on autocatalytic reaction networks. Input is a food set of molecule types and a list of catalyzed reactions (Fig. 1). It provides several fast and exact algorithms for calculating auto-catalytic subnetworks, including maximal CAFs, RAFs, and pseudo-RAFs. It allows the identification of minimal RAFs and minimal subnetworks that are both autocatalytic and able to produce specific products such as amino acids (Huson *et al.* 2024). It can also efficiently compute a unique minimal “core” RAF if one exists.

For networks with up to 250 reactions, the software provides a graphical representation and can animate molecules flowing through the network, showing how subnetworks arise. In this multi-window application, each input dataset is displayed in its own window, together with all associated analyses. The software can handle complex catalysis rules (Boolean expressions) and supports inhibition and also non-catalyzed reactions.

Running the CAF, RAF, and pseudo-RAF algorithms on the toy example shown in Fig. 1 reveals that this system does not have a CAF, but does have a maximal RAF, consisting of reactions r1, r2, and r3, and that the whole system is a pseudo-RAF.

**Figure 2.**
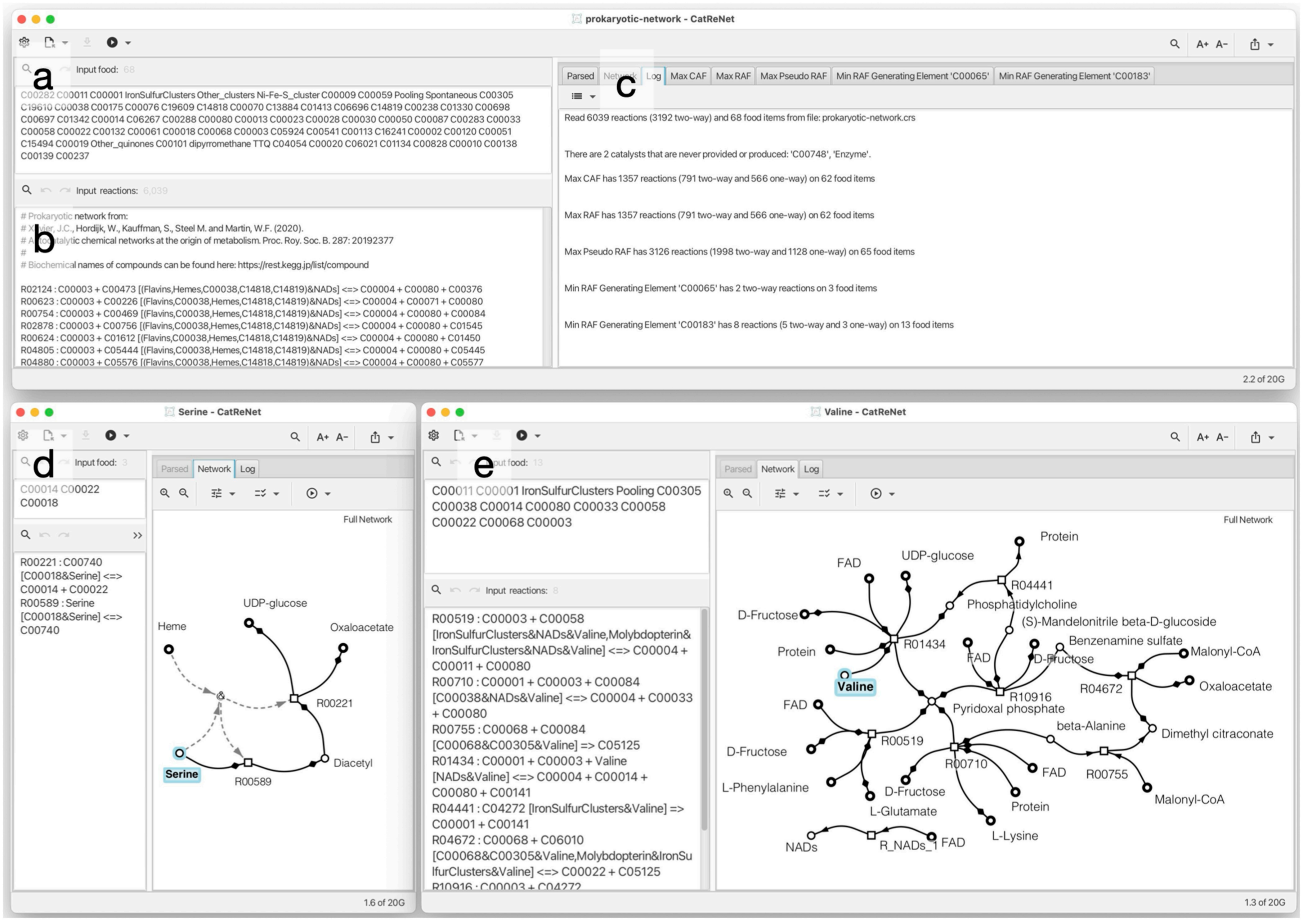
CatReNet user interface. For a large prokaryotic catalytic reaction network (Xavier *et al.* 2020), we show (a) the food input tab (68 items), (b) the reactions input tab (6039 reactions), and (c) the log tab summarizing the results of several computations. In (d) and (e) we show the extracted minimum autocatalytic subnetworks (RAFs) that generate the amino acids serine and valine, respectively.

Xavier *et al.* (2020) investigated the enzymatic and spontaneous reactions charted in modern metabolism and used RAFs as a filter to uncover elements with self-organizational properties. They provide a prokaryotic catalytic reaction network with 68 food items and 6039 reactions, of which 3192 are two-way reactions, used as input in Fig. 2a–c. One interesting application of CatReNet on this dataset is to extract the minimal autocatalytic subnetworks that are required to produce various amino acids, such as serine and valine, depicted in Fig. 2d and e, respectively (Huson *et al.* 2024). This can be addressed using the above-mentioned algorithm that takes as input a catalytic reaction network and a target set of desired products and determines a minimal RAF that produces the target set.

## 3 Conclusion

CatReNet allows researchers to interactively explore the mathematical properties of self-sustaining catalytic reaction networks. These can be used to model laboratory-scale systems (Xavier *et al.* 2020). Other areas of application include ecological and economic modeling (Cazzolla Gatti *et al.* 2020). They can also be used to investigate aspects of early life and so perhaps help address one of the most fundamental scientific challenges, the origin of life on Earth (Lane and Xavier 2024).
